# Study on the protective effects and mechanisms of eleutherococcus senticosus on korsakoff syndrome

**DOI:** 10.3389/fnins.2025.1670516

**Published:** 2025-09-19

**Authors:** Shan Jiang, Yaoai Wang, Yifan Ren, Xiaoran Sun, Jiaxin Ding, Siju Lou, Xueke Bai, Xin Hai, Galina Ramenskaya, Ning Zhang

**Affiliations:** ^1^School of Pharmacy, Heilongjiang University of Chinese Medicine, Harbin, China; ^2^Department of Pharmacy, The First Affiliated Hospital, Harbin Medical University, Harbin, China; ^3^Department of Pharmaceutical and Toxicological Chemistry Named after Arzamastsev of the Institute of Pharmacy, I.M. Sechenov First Moscow State Medical University, Moscow, Russia

**Keywords:** korsakoff syndrome, cognitive dysfunction, eleutherococcus senticosus, central nervous system, alcohol

## Abstract

Korsakoff syndrome (KS), as a central nervous system disorder caused by prolonged alcohol exposure, is primarily characterized by cognitive dysfunction, which has long-term effects on patients’ lives, and there is currently a lack of effective therapeutic drugs. Eleutherococcus senticosus (ES), as a traditional medicinal plant, has significant antioxidant, anti-inflammatory, and central nervous system protective effects, and is widely used as ethnopharmacological agen. This article elaborates on the main pathogenesis and the latest research progress of KS, summarizes the mechanisms of central nervous system protection by ES and its active components, and explores its main mechanisms and targets for treating KS, aiming to provide drug options for the effective treatment of KS while promoting the development and utilization of the medicinal value of ES.

## 1 Introduction

Korsakoff syndrome is one of the distinctive symptoms of chronic alcoholism (Pandey et al., 2017). It typically takes more than 10 years from the onset of drinking to the manifestation of chronic alcoholism symptoms (Campanella et al., 2009). Chronic alcoholism causes damage to the central nervous system and has become a prominent public health issue in Northeast China and Russia. According to 2016 statistics, nearly 8.6% of men and 1.7% of women exhibited characteristics indicative of chronic alcoholism (Rehm and Shield, 2019). However, only a small proportion of patients seek medical attention in a timely manner. KS is characterized by specific cognitive impairments, such as anterograde and retrograde amnesia, limited learning capacity, and deficits in executive functions. These impairments lead to compromised abilities in judgment, planning, and problem-solving, as well as a weakened inhibitory control of the central nervous system (Oudman et al., 2015). KS has been scarcely reported domestically, and its significance is often underestimated by patients. This has led to an increasing number of comorbidities, ultimately resulting in irreversible damage to the nervous system. In the prevention and treatment of alcohol-induced neurological damage, there has been little satisfactory progress. Therefore, there is an urgent need for new and more practical prevention and treatment strategies. KS is often more prevalent among the aging population, and cases involving brain and nervous system damage may require long-term treatment (Kunkle et al., 2019). Long-term alcohol abuse in KS patients not only leads to persistent vomiting but also causes abnormal glucose metabolism, resulting in the accumulation of pyruvic acid and lactic acid, as well as the depletion of vitamin B1. Additionally, it inhibits the intestinal absorption of vitamin B1, making conventional thiamine treatment highly limited. In addition, thiamine supplementation is limited, some patients respond poorly to treatment, necessitating the exploration of new neuroprotective agents to alleviate symptoms or slow disease progression (Muley et al., 2022).

Eleutherococcus senticosus, also referred to as Siberian ginseng, is the dried rhizome of the plant ES (Rupr. et Maxim.) Harms, belonging to the Araliaceae family. It is primarily distributed in northeastern China, Russia, and South Korea (Tong et al., 2025). Modern pharmacological studies have shown that the saponins, coumarins, flavonoids, and polysaccharides abundantly present in ES exhibit pharmacological effects including anti-tumor, antioxidant, anti-aging, anti-inflammatory, and liver-protective properties. Studies have shown that the active ingredients rich in ES possess pharmacological effects that protect the central nervous system and have significant potential in improving cognitive dysfunction (Liu et al., 2018). The abundant eleutheroside B and E in ES may improve cognitive dysfunction in aged rats by accelerating the synthesis of acetylcholine in hippocampal neurons through the activation of cholinesterase or the enhancement of choline recycling (Huang et al., 2013). Pharmacokinetic results revealed that components such as hyperoside, quercetin, quercitrin, and caffeic acid in ES can cross the blood-brain barrier, thereby achieving therapeutic effects for ischemic stroke (Wang Y. et al., 2020). The polysaccharide component of ES can significantly enhance brain’s antioxidant stress capacity, while simultaneously inhibiting the excessive levels of pro-inflammatory cytokines (IL-1β), thereby achieving the effect of protecting brain tissue in rats with brain injury models (Xie et al., 2015). Given the significant central nervous system protective effects of KS, as well as its anti-inflammatory and cognitive impairment alleviating effects, we hypothesize that ES possesses substantial pharmacological potential for treating KS.

Patients with KS experience direct/indirect neuronal damage due to long-term alcohol consumption. Alcohol can interfere with the normal metabolism of neurons, leading to mitochondrial dysfunction, disturbances in energy metabolism, and decreased stability of cell membranes. Acetaldehyde and other oxidative byproducts generated during alcohol metabolism further exacerbate neuronal damage. These metabolites trigger oxidative stress responses, leading to the production of free radicals that damage lipids, proteins, and deoxyribonucleic acid (DNA) in nerve cells (Koike and Sobue, 2006). Long-term alcohol intake can also activate microglia, which release pro-inflammatory factors, thereby exacerbating neuroinflammation and neuronal damage (Nutt et al., 2021). Disruption of oligodendrocyte function can also lead to neuronal damage. The primary role of oligodendrocytes is to generate the myelin sheath that wraps around neuronal axons. The myelin sheath serves to cut off and accelerate the transmission of nerve impulses, enhancing efficiency of neural signal transmission. Additionally, oligodendrocytes can produce lactate to provide energy support for neurons (Kuhn et al., 2019). Alcohol exposure inhibits the proliferation and differentiation of oligodendrocyte precursor cells (OPCs), affecting oligodendrocyte generation and leading to a reduction in the number of mature oligodendrocytes, thereby impairing myelination (Huang et al., 2024). Damage to the myelin sheath can lead to a slowdown in neuronal conduction speed, affecting the normal function of neurons (Darbinian and Selzer, 2022), thereby leading to memory impairment, cognitive decline, and motor coordination disorders.

In summary, direct/indirect neuronal damage, increased neuroinflammation, and elevated oxidative stress levels are the core pathological features of KS, and the antioxidant, anti-inflammatory, and neuroprotective effects of ES may directly intervene in these pathological characteristics.

## 2 Current research progress and pathogenesis of korsakoff syndrome

### 2.1 The current research progress of KS

Approximately 2 billion people worldwide consume alcohol, and chronic alcohol abuse can lead to various diseases and death. Globally, 3 million people die each year from alcohol consumption. A survey from the China Kadoorie Biobank study found that 33% of hospitalization incidents among Chinese men are closely related to alcohol intake (Im et al., 2023). In recent years, the impact of alcohol on the significant fluctuations in mortality rates in Russia has now been widely recognized (McKee, 1999). Alcohol is a significant factor contributing to premature deaths among Russians, with both the quantity and manner of consumption being detrimental to health (Neufeld and Rehm, 2013). As the seventh major risk factor for global mortality and handicapped, the risk of developing cognitive dysfunction after alcohol cessation remains unchanged (Schwarzinger et al., 2018).

Some alcoholics may have a genetic predisposition to develop KS (Arts et al., 2017). Its characteristic neuropathology includes neuronal loss, microhemorrhages, and gliosis in the periventricular gray matter and periaqueductal gray matter of the midbrain (Kopelman et al., 2009). Alcohol and its metabolite acetaldehyde possess direct neurotoxicity. Furthermore, chronic alcoholics often develop severe liver diseases, which themselves can lead to alterations in thiamine deficiency, cognitive dysfunction, and central nervous system injury (Butterworth, 1995). A small number of patients can develop KS without alcohol consumption, such as those with AIDS, terminal cancer, or chronic infections and malnutrition (Popa et al., 2021).

Patients with KS often suffer from severe physical discomfort and mental illness, progressing from initial symptoms of amnesia, confabulation, paramnesia, cognitive dysfunction, and disorientation, ultimately leading to changes in personality. The various health issues of these patients severely limit their ability to perform daily activities and have a negative impact on their social functioning (van Dam et al., 2020). Therefore, effective treatment for KS is of great importance.

### 2.2 The pathogenesis of KS

#### 2.2.1 Alcohol directly damages neuronal cells and induces mitochondrial dysfunction

Mitochondria are the organelles that provide the energy required for the brain to maintain neuronal communication, and their dysfunction is considered a major factor in alcohol-induced synaptic dysfunction (Mira et al., 2019). In various affected organizations, the enzyme required for oxidizing ethanol produces acetaldehyde, which is then converted to acetate by aldehyde dehydrogenase (ALDH). ALDH is a nicotinamide adenine dinucleotide (NAD^+^)-dependent enzyme, and mitochondrial ALDH2 is likely the primary contributor to the clearance of ethanol-derived acetaldehyde in cells. Alcohol metabolism has several adverse effects on mitochondria, including elevated levels of free radicals, hyperacetylation of mitochondrial proteins, and excessive mitochondrial fragmentation (Waddell et al., 2022). Prolonged exposure to ethanol activates the mitochondrial fission protein dynamin-related protein 1 (Drp1) and upregulates Drp1 receptors such as fission protein 1 (Fis1), mitochondrial dynamics protein of 49 kDa (Mid49), and mitochondrial fission factor (Mff), while reducing the levels of optic atrophy 1 (Opa1) and mitochondrial fusion protein mitofusin 1 (Mfn1). The mitochondrial division inhibitor 1 (mdivi-1) eliminates ethanol-induced mitochondrial homeostasis imbalance and improves cognitive dysfunction in the mouse model. By inhibiting the abnormal activation of cyclin-dependent kinase 5 (Cdk5), it is possible to mitigate hippocampal neuronal damage and cognitive dysfunction induced by mitochondrial fission and mitochondrial homeostasis imbalance mediated by mitochondrial fission proteins Drp1 due to prolonged exposure to ethanol (Liu et al., 2022).

**FIGURE 1:**
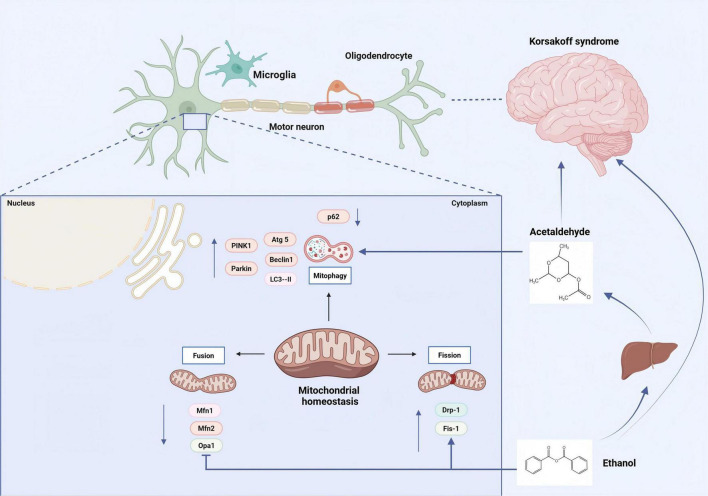
Alcohol and its metabolite ethanol directly damage neuronal cells, leading to mitochondrial dysfunction and the pathogenesis of KS. Alcohol metabolism has several adverse effects on mitochondria, including elevated levels of free radicals, hyperacetylation of mitochondrial proteins, and excessive mitochondrial fragmentation. [Arrows (→) indicate positive regulations, and the symbol (T) represents negative regulations].

After excessive alcohol consumption, acetaldehyde, the most toxic metabolite of ethanol, impairs mitochondrial function and induces cytotoxicity in neuronal cells. In acetaldehyde-treated cells, the levels of light chain 3 (LC3)-II, Beclin1, autophagy-related proteins (Atg) 5 and Atg16L1, PTEN-induced putative kinase (PINK) 1, and Parkin were significantly increased, while the level of p62 was decreased. Acetaldehyde significantly increased the accumulation of PINK1 and Parkin on mitochondria, accompanied by a decline in mitochondrial quality. Hyperactive mitophagy may be a core mechanism underlying acetaldehyde-induced neuronal cytotoxicity. The antioxidant N-acetyl-L-cysteine remarkably attenuates the mitophagy response and alleviates acetaldehyde-induced cytotoxicity, suggesting that oxidative stress is the primary intermediary of acetaldehyde-induced intense mitophagy (Yan et al., 2022). The specific mechanism is illustrated in Figure 1.

#### 2.2.2 Alcohol directly damages neuronal cell death and leads to synaptic injury regeneration disorder

Alcohol abuse triggers neuroinflammation, leading to neuronal damage and further memory and cognitive impairment (Guergues et al., 2020). Stereotypic patterns of synaptic connections between neurons underlie the ability of the CNS to perform complex but circuit-specific information processing (D’Orazi et al., 2016). The loss of excitatory synapses is known to form the cognitive impairment in AD patients (Bhembre et al., 2023). Research has found that alcohol exposure can also lead to synaptic regeneration disorders, which in turn result in the occurrence of cognitive dysfunction behaviors. Free radicals significantly promoted the concentration of reactive oxygen and nitrogen species in the system, thereby elevating overall oxidative stress. A proper balance between free radicals and antioxidants is crucial for maintaining physiological functions. Alcohol, as the main cause of the enhancement of reactive oxygen species (ROS) in the brain, has an adverse impact on axonal regeneration and synaptic plasticity, leading to the death of neuronal cells. In addition, the increase of ROS in the brain alters a variety of signaling pathways, such as apoptosis, autophagy, inflammation and microglial activation, DNA damage response, and cell cycle arrest, thus resulting in deficits in memory and learning abilities (Tripathi et al., 2022). Ethanol may disrupt cholesterol homeostasis during brain development, thereby causing synaptic regeneration disorders (Guizzetti and Costa, 2005).

#### 2.2.3 Alcohol-induced oligodendrocyte death and demyelination

Oligodendrocytes are myelin-forming cells of CNS, which are generated from oligodendrocyte precursor cells (OPCs) that express neurotransmitter receptors (Lu et al., 2023). Their main function is to wrap around axons to form a shielding myelin sheath structure, thereby promoting the saltatory and efficient transmission of bioelectrical signals, and maintaining and protecting the normal functions of neurons (Bansal and Zinkus, 2019). Chronic ethanol intoxication can induce oxidative stress and neuroinflammation, partially mediated by platelet endothelial cell adhesion molecule 1 (PECAM-1). Alcohol intoxication may lead to BBB damage, bringing about demyelination of oligodendrocytes and cognitive dysfunction. The increased expression of oligodendrocytes and myelin-related proteins has been associated with improvements in certain cognitive function impairments in various aspects (Lu et al., 2024). These changes and the upregulation of PECAM-1 may constitute the pharmacological mechanism of recovery in ethanol-induced oxidative damage. Activation of calpain, a calcium-activated neutral protease, has been found to cause detrimental alterations in spinal motor neurons following *in vitro* exposure to ethanol. Studies have shown that axonal proteins and myelin basic protein are significantly reduced in multiple brain regions after alcohol intoxication (Hyatt et al., 2022). Calpain inhibitors can markedly protect the ultrastructural integrity of oligodendrocytes, and it has been confirmed that calpain inhibitors serve as protective agents against central nervous system neurodegeneration (Samantaray et al., 2015). Ethanol can influence microglia in the brain, indirectly altering myelination. Activation of the glial cell Toll-like receptor 4 (TLR4) can activate microglia, resulting in oligodendrocyte death and demyelination. Specifically, Research has found that ethanol activates TLR4, which is involved in reducing the expression of proteins related to myelination, including proteolipid protein (PLP), myelin basic protein (MBP), myelin oligodendrocyte glycoprotein, 2′,3′-cyclic nucleotide 3′-phosphodiesterase, and myelin-associated glycoprotein. However, in TLR4 receptor knockout mice, most of the alterations in myelin were not directly observed (Alfonso-Loeches et al., 2012).

#### 2.2.4 Alcohol-induced microglial activation leads to neuroinflammation

**FIGURE 2:**
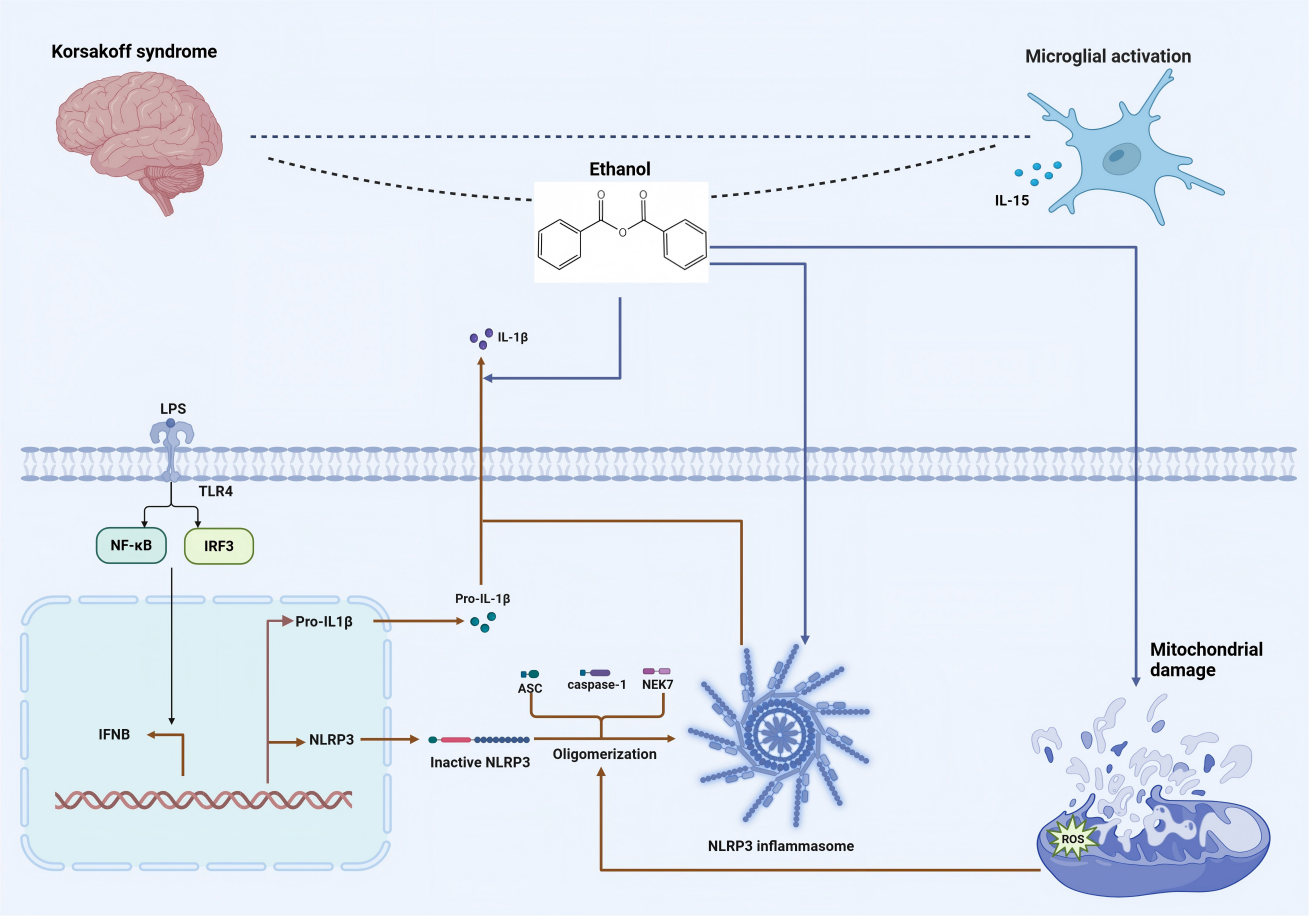
The mechanism by which alcohol induces the activation of the NLRP3 inflammasome, resulting in the activation of microglia and the pathogenesis of KS. Alcohol exposure increases the initiation of the NLRP3 inflammasome, enhancing the secretion of cytokines by activated microglia. [Arrows (→) indicate positive regulations, and the symbol (T) represents negative regulations].

Microglia are the resident immune cells in the brain and are capable of showing a wide variety of activation phenotypes. Many of these phenotypes are associated with various diseases of the central nervous system, including those related to chronic alcohol abuse (Guergues et al., 2020). Microglial cells are believed to play the role of the brain’s resident immune defense system (Poh et al., 2019). As representatives of macrophages in CNS, microglial cells have the ability to clear cellular debris. The signaling of microglial cells is one of the main targets of the action of ethanol in the brain: Exposure to ethanol selectively modulates the intracellular signaling in microglial cells instead of broadly suppressing signaling pathways in a non-specific way. The dysregulation of the inflammatory activation signaling of microglial cells induced by ethanol may lead to disorders of the immune and inflammatory responses in CNS (Suk, 2007). The activation of microglia leads to the release of pro-inflammatory mediators, and microglia-mediated neuroinflammation has been recognized as one of the neuropathological mechanisms induced by alcohol. Interleukin-15 (IL-15) is a cytokine, and its expression will increase when astrocytes and microglia are activated under the condition of long-term alcohol exposure (Waldmann et al., 2020). Importantly, the partial activation of microglia induced by ethanol cannot be reversed by prolonged abstinence. Chronic alcohol exposure induces a microglial phenotype consistent with partial activation, without a significant increase in the classical cytokine markers of neuroinflammation in hippocampus. Moreover, abstinence after long-term alcohol abuse is insufficient to change the ethanol-induced partially activated phenotype of microglia (Cruz et al., 2017). Alcohol exposure can lead to cognitive dysfunction in mPFC and excessive activation of the NLRP3 inflammasome. Adolescent ethanol exposure triggers microglia-mediated neuroinflammation through TLR4 activation, while SUMO-specific protease 6 (SENP6) is crucial for inhibiting NF-κB pathway activation and neuroinflammation (Li et al., 2019). The excessive activation of microglia is the main cause of cognitive dysfunction. Alcohol promotes mitochondrial dysfunction and the generation of ROS, and accelerates the excessive activation of the NLRP3 inflammasome, leading to the production of inflammatory factors. Alcohol exposure increases the initiation of the NLRP3 inflammasome, enhancing the secretion of cytokines by activated microglia. Excessive alcohol intake will cause damage to mPEC, thus leading to cognitive dysfunction (Li J. et al., 2023). Following alcohol exposure, an increase in the synthesis and excretion of AGE albumin was also observed in activated microglia. AGE-albumin significantly increases the expression levels of receptor for AGE (RAGE)-positive neurons via the mitogen-activated protein kinase pathway. This elevation in RAGE expression also exacerbates RAGE-dependent neuronal death. However, in animal models, treatment with soluble RAGE or AGE inhibitors can notably reduce the neuronal damage caused by AGE, suggesting that AGE has a detrimental effect on neurons through the activation of the RAGE pathway, and interventions targeting this pathway can mitigate such damage. The increase in AGE albumin in activated microglia induces neuronal cell death, thereby leading to cognitive dysfunction (Byun et al., 2014). The specific mechanism is illustrated in Figure 2.

#### 2.2.5 Alcohol induces cognitive dysfunction through the gut-brain axis

Alcohol consumption has a direct impact on the gut microbiota, altering bacterial diversity and leading to bacterial overgrowth. Increasing evidence suggests that the effects of alcohol on the gut microbiome may contribute to increased the development of alcohol-related diseases such as KS (Wolstenholme et al., 2024). Alcohol use impairs the intestinal barrier and causes changes to the intestinal permeability as well as the gut microbiota composition (Wang S. et al., 2020). Chronic alcohol abuse leads to the overactivation of enteric glial cells in addition to the excessive activation of microglia (Khan and Chang, 2023). Therapeutic interventions targeting the gut-brain axis as a novel strategy for treating KS-associated cognitive dysfunction (Li et al., 2022). The gut-brain axis harbors an abundance of peptides, such as the orexigenic peptide ghrelin. In animal models, agonists of the GLP-1 or amylin receptor and ghrelin receptor (GHSR) antagonists reduce alcohol drinking, relapse drinking, and alcohol-seeking (Tufvesson-Alm et al., 2022). After prolonged alcohol abuse, alcohol not only damages the intestinal barrier in KS patients but also further affects the brain via the gut-brain axis, leading to occurrence of cognitive dysfunction. By restoring the intestinal barrier and inhibiting alcohol metabolism, the goal of treating KS is achieved (Wu et al., 2024).

After alcohol withdrawal, the concentrations of tryptophan metabolites, norepinephrine, dopamine, and γ-aminobutyric acid in the prefrontal cortex and hippocampus are related to the changes in bacterial abundance, and this relationship is related to gender (Pizarro et al., 2021). In the rat model induced by alcohol, changes occurred in intestinal permeability and blood metabolic levels. Biosynthetic pathways such as those of valine, leucine, and isoleucine, as well as the metabolism of arginine and proline, were affected by alcohol (Yang et al., 2021). The extracellular vesicles secreted by the gut microbiota mainly affect the central nervous system through the gut-brain axis (Pei et al., 2024). Fecal microbiota transplantation may alter the gut microbiota, improve intestinal permeability, and reduce the inflammatory response, thereby treating the cognitive dysfunction behaviors caused by alcohol (Docherty, 2025).

## 3 Neuroprotective mechanisms of ES in KS

In traditional medicine, ES is an effective medicinal plant for the treatment of hypertension, thrombosis, inflammation and cancer (Wu et al., 2019). As a typical medicinal plant in the northeastern region of China, it is primarily distributed in Heilongjiang, Jilin, Liaoning, and Hebei provinces (Wang et al., 2014). Research has found that it is rich in chemical components such as eleutheroside, phytosterols, triterpenoid saponins, dihydrodehydrodiconiferyl alcohol monopyranoside, glycosides, 5′-O-caffeoylquinic acid isomers, glucopyranosides, and lignans (Deyama et al., 2001). As a medicinal plant traditionally used in Far Eastern Russia and East Asian medicine, ES is classified as an adaptogen-a category of herbal products with non-specific, systemic anti-stress effects that impact the entire human body. Initially recognized as a medicinal plant in the pharmacopeia of the USSR in 1962, it is currently recommended by the European Medicines Agency for the treatment of conditions such as fatigue and weakness. A safe and well-tolerated herbal remedy, it is suitable for use by adults, children, and pregnant women (Gerontakos et al., 2021).

### 3.1 ES possesses clinical application and efficacy advantages in targeted treatment of central nervous system diseases

Eleutherococcus senticosus possesses pharmacological effects including anti-fatigue, anti-stress, immune enhancement, central nervous system activity, and antidepressant properties (Chen et al., 2023), Rich in active ingredients in addition saponins, flavonoids, lignans, and polysaccharides (Ma et al., 2025). Clinically, ES is commonly used to improve nerve damage and treat conditions such as insomnia and depression (Wu et al., 2025). Lee et al. (2017) discovered that the ethanol extract of Acanthopanax improves cognitive dysfunction induced by cholinergic blockade by modulating memory-related signaling molecules. ES can effectively alleviate behavioral symptoms in PD mice, reduce oxidative stress in hippocampal neurons of brain tissue, and is closely related to pathways such as glutathione metabolism and glutamate-aspartate and glutamate metabolism (Fu et al., 2024). ES extract has the effect of alleviating reperfusion injury in neuronal cells following oxygen-glucose deprivation, and it has been demonstrated to protect neural cells by inhibiting oxidative stress, preserving mitochondrial homeostasis, and suppressing cell apoptosis (Wang et al., 2022). The extract of ES effectively controlled the neuronal cell swelling, protein loss, and liquefaction of necrotic tissue observed in the brains of radiotherapy model mice (Zhou A. et al., 2018). Sixty active substances in ES be recognized using the UPLC-Q-TOF-MS method. The research findings of systems pharmacology have revealed that ES treats cognitive impairment through the acetylcholinesterase and apoptosis signaling pathways. Fifteen potential anti-AD components in ES protect against cholinergic nervous system damage and reduce scopolamine-induced neuronal apoptosis (Zhuo et al., 2023). The aqueous extract of ES leaves exerted a beneficial effect on the restoration of neurite outgrowth in Aβ25-35-induced degeneration, as determined by axonal density measurement. The findings demonstrated that compounds 2 and 3 in the aglycone inhibited Aβ25-35-induced degeneration and significantly rehabilitated axonal growth (Ge et al., 2016).

### 3.2 Potential effective components for the treatment of KS exist in ES

#### 3.2.1 The active components of Acanthopanax senticosus target and inhibit neuronal cell apoptosis

As a psychoactive substance widely used worldwide, the excessive use of alcohol is bound to cause cognitive impairment and central nervous system damage (García-Baos et al., 2021). Alcohol affects the activity of neuronal circuits (Egervari et al., 2021). Identifying the molecular effects within specific neurons in brain regions that lead to behavioral changes during acute and chronic ethanol exposure is one of the core aspects in treating KS (Abrahao et al., 2017). The drinking behavior of pregnant women can cause continuous damage to hippocampal neurons and a decrease in the mass of the hippocampus (Burke et al., 2015). Long-term alcohol exposure leads to a significant decrease in thiamine levels in the body. Alcohol-induced decline in thiamine levels is closely associated with two mitochondrial pathways (Bâ, 2017).

Eleutheroside B is an active ingredient extracted from the roots and stems of ES, which has neuroprotective effects (Chiarugi, 2023). It exerts neuroprotective effects against hypoxia/reoxygenation-induced injury in cortical neurons of SD rats, and the underlying mechanism might be related to promoting the activation of the PI3K/Akt signaling channel to inhibit neuronal apoptosis, reduce the degree of lipid peroxidation, and enhance activity index of antioxidant enzymes (Deng et al., 2020). Eleutheroside B enhances the interaction between p53 apoptosis-stimulating protein inhibitor (iASPP) and Keap1, stabilizes Nrf2 levels in the brains of APP/PS1 mice, inhibits p53 DNA-binding activity, reduces oxidative stress in the brain, decreases Aβ accumulation and neuronal apoptosis, improves hippocampal synaptic plasticity, and thereby alleviates cognitive deficits in APP/PS1 mice (Ding et al., 2022). The triterpenoid saponins in ES can significantly reduce β-amyloid-induced neural network damage by promoting the growth of neurites and axons (Zhou et al., 2023). Eleutheroside E (EE), the primary component of ES, exhibits significant protective effects on cognitive function. It alleviates cognitive dysfunction induced by isoflurane anesthesia through the modulation of the α7-nAChR-NMDAR signaling pathway (Lu and Xiao-Qing, 2019). The active components of ES inhibit neuronal apoptosis, reduce oxidative stress in the brain, and alleviate cognitive dysfunction.

The main flavonoid components of ES include hyperoside, rutin, quercetin, and quercitrin. The primary organic acid components consist of caffeic acid, 1,5-dicaffeoylquinic acid, 4,5-dicaffeoylquinic acid, and 3,4-dicaffeoylquinic acid (Wang et al., 2019). The flavonoid components in ES, such as quercetin and quercitrin, can alleviate lipid peroxidation damage, enhance the expression of Bcl-2 protein, and reduce neuronal apoptosis in the ischemic brain region, thereby protecting the damaged brain tissue (Zhu et al., 2012). Mice subjected to chronic ethanol treatment exhibited poor memory retention in the step - down passive avoidance behavior and elevated plus maze tasks. Quercetin has remarkable antioxidant activity. After long-term therapeutic administration, the cognitive behaviors of mice in the aging model and the alcohol disorder model have been improved (Singh et al., 2003). Quercetin can treat the cognitive impairment behaviors and AD pathological manifestations in APP/PS1 double transgenic mice. Its mechanism of action is closely related to the activation of the Kelch-like ECH-associated protein 1 (Keap1)/nuclear factor erythroid 2-related factor 2 (Nrf2)/heme oxygenase-1 (HO-1) signaling pathway and the inhibition of apoptosis by quercetin (Cheng et al., 2024). The flavonoid components of Acanthopanax activate the Keap1/Nrf2/HO-1 signaling pathway, reduce neuronal cell apoptosis, protect damaged brain tissue, and improve cognitive dysfunction.

**FIGURE 3:**
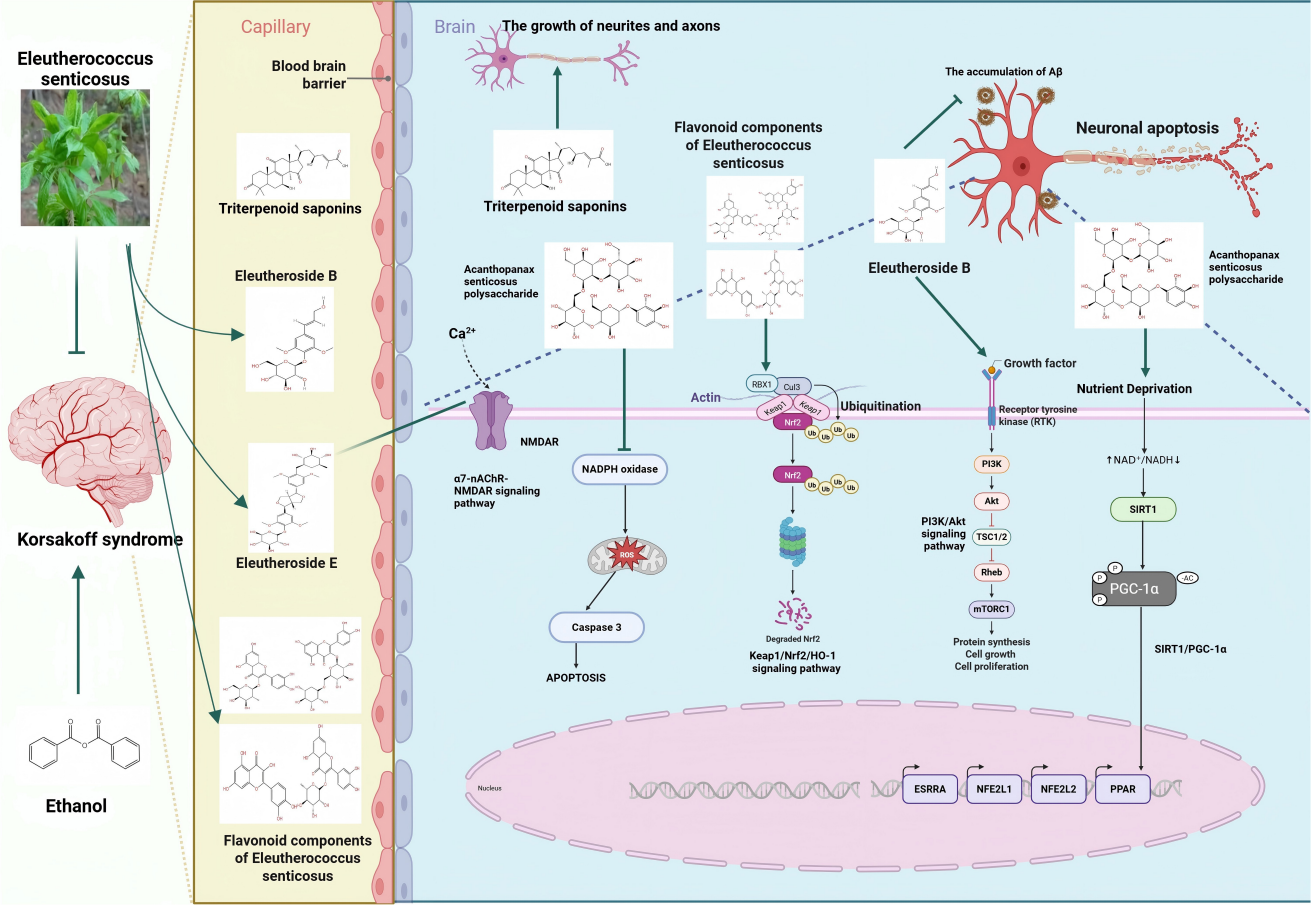
Inhibition of neuronal damage by active components of eleutherococcus senticosus. [Arrows (→) indicate positive regulations, and the symbol (T) represents negative regulations].

The polysaccharide active ingredients in ES significantly mitigated the alterations in glucose and C-reactive protein (CRP) levels caused by alcohol, thereby reducing the severity of alcohol hangover through inhibition of alcohol-induced hypoglycemia and neuronal inflammatory responses (Bang et al., 2015). Acanthopanax polysaccharide can protect against H_2_O_2_-induced apoptosis in hippocampal neurons by downregulating release level of pro-apoptotic protein caspase-3 and enhancing the activity of DNA damage repair enzymes, thereby inhibiting ROS-induced neuronal apoptosis (Liu et al., 2013). Acanthopanax polysaccharides can ameliorate neural damage in SD rats, potentially through the activation of the SIRT1/PGC-1α pathway, enhancing SOD activity in brain tissues, reducing MDA content, improving mitochondrial function and oxidative stress, ultimately alleviating ischemic brain injury. Activation of the SIRT1/PGC-1α pathway can improve mitochondrial damage and reduce ROS production, inhibit neuroinflammation, thereby improving cognitive function impaired by chronic cerebral hypoperfusion (Dai et al., 2025). The polysaccharide component of ES inhibits mitochondrial dysfunction, protects neuronal cells, suppresses their apoptosis, and alleviates cognitive dysfunction. The specific mechanism is illustrated in Figure 3 and Table 1.

**TABLE 1:** The specific mechanism by which eleutherococcus inhibits neuronal apoptosis.

Eleutherococcus senticosus components	The mechanism of action for treating KS
Eleutheroside B	1. Activate the PI3K/Akt signaling pathway 2. Inhibit p53 DNA-binding activity 3. Reduce oxidative stress in the brain 4. Decrease Aβ accumulation and neuronal apoptosis 5. Improve hippocampal synaptic plasticity
Eleutheroside E	1. Modulate the α7-nAChR-NMDAR signaling pathway
Flavonoid components	1. Alleviate lipid peroxidation damage 2. Enhance the expression of Bcl-2 protein 3. Educe neuronal apoptosis 4. Activate the Keap1/Nrf2/HO-1 pathway
Polysaccharide active ingredients	1. Inhibite alcohol-induced hypoglycemia and neuronal inflammatory responses 2. Activate of the SIRT1/PGC-1α pathway 3. Improve mitochondrial damage 4. reduce ROS production 5. inhibit neuroinflammation

#### 3.2.2 The active components of Acanthopanax senticosus target and inhibit microglial activation

Neuroinflammation triggered by microglial activation and the consequent neurological dysfunction are prominent features of cognitive impairments such as KS (Li Q. et al., 2023). Activation of microglia in the prefrontal cortex predicts cognitive decline in frontotemporal dementia (Malpetti et al., 2023). Mitophagy is a process that regulates inflammatory responses by limiting the accumulation of damaged mitochondria. Inducing microglial mitophagy may be an effective therapeutic approach for KS patients (Zhang et al., 2025). Under alcohol exposure conditions, the activation of hippocampal microglia significantly increases (Walter et al., 2023). The specific transcriptome of microglia undergoes changes after long-term alcohol consumption (McCarthy et al., 2018). Repeated ethanol exposure potentiates microglial activity (Marshall et al., 2016). Microglia are critical regulators of alcohol responses in the CNS (Henriques et al., 2018). That early EtOH exposure caused a deficit in experience-dependent synaptic plasticity in the visual cortex (Wong et al., 2018).

**FIGURE 4:**
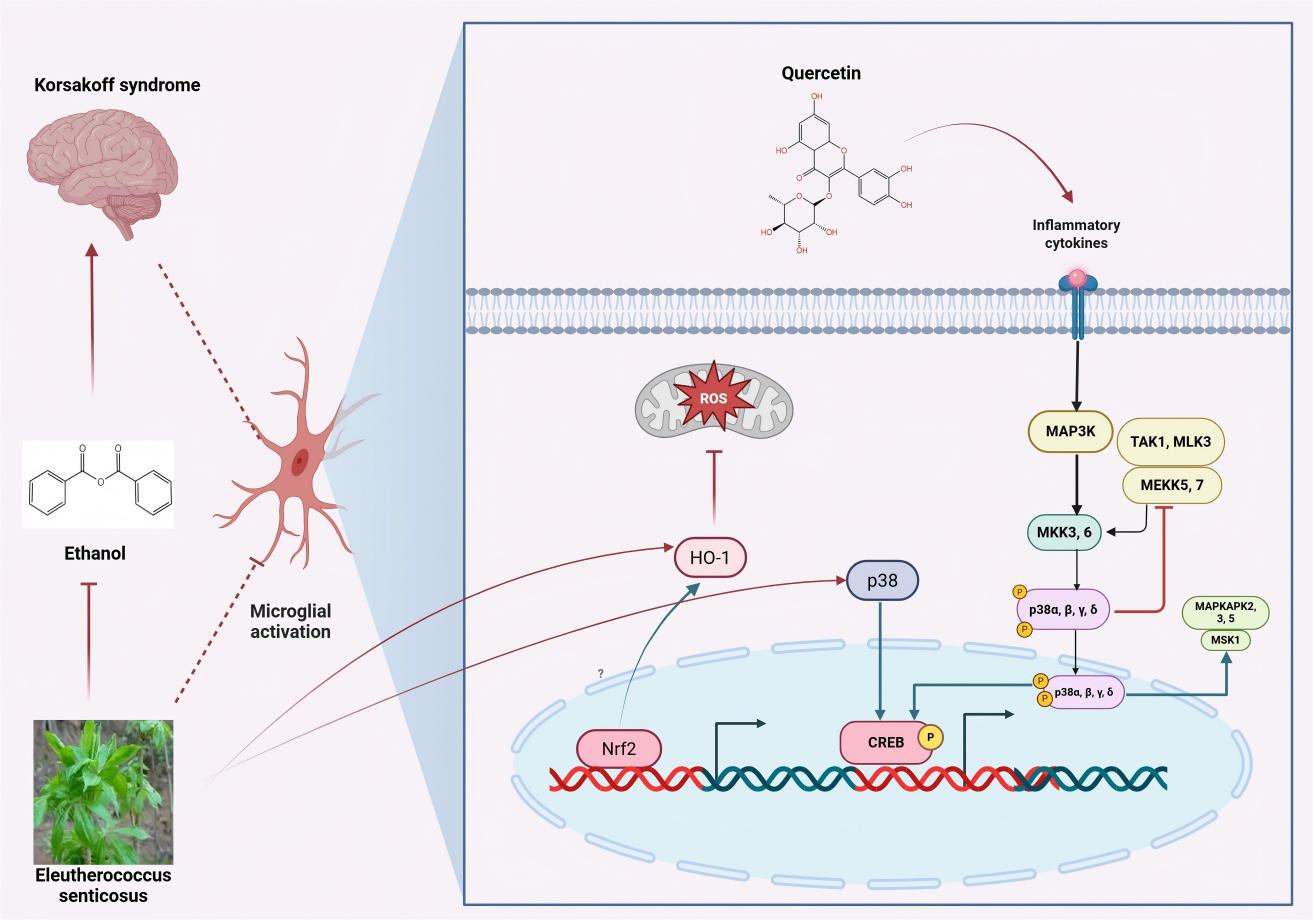
Inhibition of microglial activation by active components of eleutherococcus senticosus. The aqueous extract of ES fruits increases HO-1 expression and reduces NO/ROS production in LPS-induced microglial cells. ES and its active components activate the p38-CREB pathway in microglia and promote the phosphorylation of the MAPK signaling pathway. [Arrows (→) indicate positive regulations, and the symbol (T) represents negative regulations].

As an inducible enzyme in cells, the aqueous extract of ES fruits increases HO-1 expression and reduces NO/ROS production in LPS-induced microglial cells. Furthermore, the induction of HO-1 expression protects cells from glutamate-induced neuronal cell death. The activation of the p38-CREB pathway and the translocation of Nrf2 are closely associated with ES-induced HO-1 expression. ES inhibits neuroinflammation and protects the central nervous system through the p38-CREB pathway-induced HO-1 expression. Additionally, ES increases the translocation of Nrf2 to regulate HO-1 expression (Jin et al., 2013). The quercetin component in Acanthopanax senticosus exerts therapeutic effects on cognitive dysfunction in AD patients by promoting the phosphorylation of the MAPK signaling pathway in microglia (Zhang et al., 2024). ES and its active components activate the p38-CREB pathway in microglia and promote the phosphorylation of the MAPK signaling pathway, thereby inhibiting neuroinflammation and achieving therapeutic effects on cognitive dysfunction. The specific mechanism is illustrated in Figure 4.

#### 3.2.3 The active ingredients of ES target the gut-brain axis

Research has found that there is a significant connection between the gut microbiota and the brain. Through this connection, the gut microbiota can further affect the CNS and brain development (Klann et al., 2021). KS is closely related to the gut-brain axis. The stability of the gut microbiota can improve cognitive dysfunction through the gut-brain axis (Pan et al., 2023). Existing studies have found that stabilizing the gut microbiota and activating the Nrf2 signaling pathway can inhibit neuroinflammation and promote neurogenesis, thereby alleviating cognitive impairment (Kong et al., 2024). Bacterial species and genera are closely related to the mediators of the microbiota-gut-brain axis, and these mediators are associated with the markers of the amyloid cascade in AD (Marizzoni et al., 2023).

Eleutherococcus senticosus regulated the gut microbiota in the ischemic stroke model, mainly by reducing the abundance of pathogenic bacteria and increasing the abundance of probiotic bacteria, such as Lactobacillus reuteri and Clostridium butyricum (Wang et al., 2023). ES extract achieves the therapeutic effect on cognitive dysfunction by altering the abundance of gut microbiota. Bacteria of the genus Ruminococcus and those of the order Clostridiales are closely related to the synthesis pathway of serotonin 5-hydroxytryptamine (5-HT). Bacteria of the genus *Streptococcus* are associated with the synthesis of 5-HT and acetylcholine (ACH). ES increased the level of tight junction proteins, inhibited the expression of inflammation in the colon, promoted the protein expression levels of brain-derived neurotrophic factor (BDNF) and NF-κB in the hippocampus of irradiated mice, and decreased the relative protein expression level of nuclear factor kappa B inhibitor alpha (IκBα) in the hippocampus (Song et al., 2023). ES exerts a therapeutic effect on ischemic stroke through the gut-brain axis and shows great potential for treating KS.

## 4 Construction of KS model

The successful model construction as the foundation for effective KS treatment. Currently, it is relatively common to use alcohol and mice to construct a KS cognitive dysfunction model (Golub et al., 2015). Human epidemiological studies suggest that the female brain may be more susceptible to the toxic effects of alcohol. Researchers constructed a cognitive impairment model with gender differences using SD rats to study the effects of alcohol exposure on gender disparities (Savage et al., 2000). In addition to conventional mouse and rat models, the latest research utilizes zebrafish for the construction of cognitive impairment models. Zebrafish exposed to alcohol exhibited impairments in retaining the memory of learned tasks (Rajesh et al., 2018). The zebrafish is currently a popular animal model in pharmacogenetics and neuropharmacology (Kalueff et al., 2014). Research has found that during the embryonic development of zebrafish, even with low-dose alcohol intake, there will be significant changes in their behavior. Alcohol has an impact on the expression of neuronal markers and the phenotypes of glial cells in the model (Paul et al., 2020).

Cognitive deficits associated with alcoholism are also a consequence of cerebellar dysfunction (Botta et al., 2010). Therefore, constructing a typical *in vitro* alcohol exposure cell model is equally essential for exploring the pathogenesis of KS and identifying effective treatment strategies. Existing studies have further dissected the characteristics of the reduced initial T cell population known to exist in alcohol-exposed mice, and have described alterations in the phenotype of effector regulatory T cells associated with the pathogenesis of chronic alcohol-induced immune dysfunction (Paterson et al., 2023). Alcohol exposure also causes changes in neuronal genes (Tyler and Allan, 2014). The co-culture technique of neurons and microglia has become the preferred *in vitro* pharmacological model for studying the effects of drugs on the behaviors associated with alcohol-induced cognitive dysfunction (Li et al., 2024).

**FIGURE 5:**
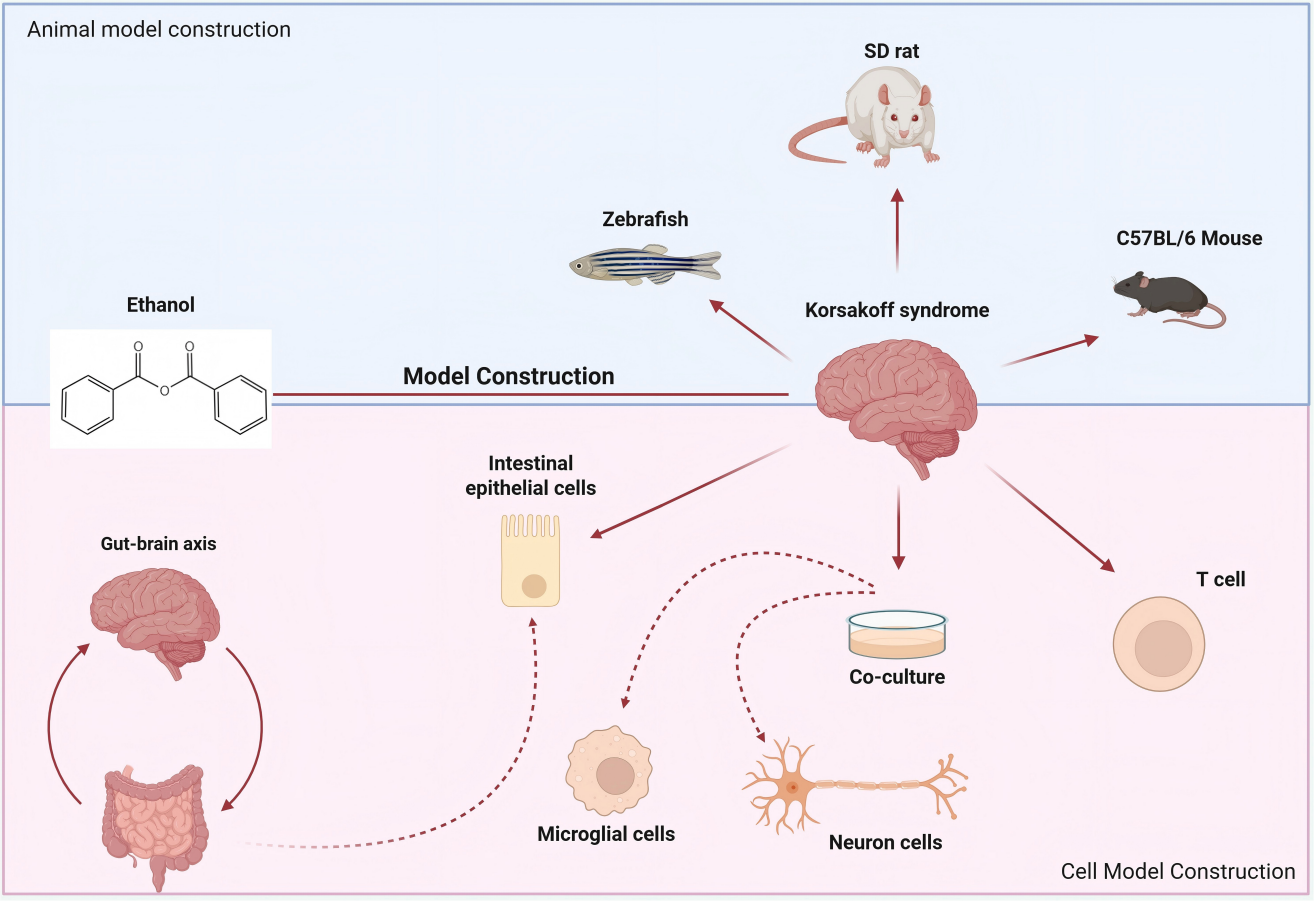
Construction of animal models and cell models related to KS. The cognitive impairment model constructed with SD rats is used to study the impact of alcohol exposure on gender differences. Zebrafish exposed to alcohol have also become an important model for studying KS.

Due to thiamine deficiency, which is a common condition associated with alcohol abuse and linked to high morbidity and mortality rates, its deficiency may lead to a rarer condition known as KS (Cook et al., 1998). After long-term alcohol consumption, it leads to insufficient nutritional intake of thiamine. Following the damage to the gastrointestinal tract, the absorption of thiamine is reduced, and there are obstacles to the bioavailability of thiamine in the body’s cells (Martin et al., 2003). Long-term excessive alcohol consumption can cause damage to the body’s intestinal barrier function. Even though the intestinal epithelial cells are renewed every 3–5 days after stopping excessive alcohol consumption, the impairment of intestinal function still persists (Lu et al., 2017). Therefore, the construction of a damaged intestinal stem cell model is also central to investigating the pathogenesis of KS in Figure 5.

## 5 Advances in pharmacokinetic studies of ES

The pharmacokinetic results of ES showed that EE was distributed in the brain tissues of irradiated mice (Song et al., 2022). The high-performance liquid chromatography combined with solid-phase extraction method was used to determine the content of isofraxidin in the plasma of rats after oral administration of Acanthopanax extract (ASE), and the pharmacokinetic behavior of isofraxidin in ASE or pure compound was also determined. The results showed that after rats were orally administered with Acanthopanax extract, the content of isofraxidin in their plasma was the sum of the contents of free isofraxidin and its precursors in the Acanthopanax extract *in vitro* (Sun et al., 2007). Eleutheroside B, as an active ingredient of traditional Chinese medicine, has a bioactivity score ranging from −0.08 to 0.38 (Ahmed et al., 2021). The changes in metabolic levels in the plasma and brain of rats after oral administration of ES extract were explored by liquid chromatography-tandem mass spectrometry (LC-MS/MS) technology. The identified active ingredients in plasma and the cerebral cortex were Eleutheroside C3, Eleutheroside M, Eleutheroside B, and Eleutheroside A1. Those three compounds as well as the leaf extract had dendrite extension activity against primary cultured cortical neurons. The effect might relate to memory enhancement (Yamauchi et al., 2019). The ultra-high performance liquid chromatography-tandem mass spectrometry of the triple quadrupole system is used to detect the contents of four triterpenoid components of ES in rat plasma and monitor its pharmacokinetic characteristics (Song et al., 2016). The proteomics results indicate that the ES extract significantly inhibits the functional changes in the expression of radiation response proteins in the PFC of mice (Zhou Y. et al., 2018). The findings from omics studies indicate that the active components in ES are significantly distributed in brain tissues, demonstrating therapeutic potential for KS.

## 6 Conclusion and outlook

The active components in ES protect neuronal cells, combat oxidative stress, preserve mitochondrial function, inhibit neuronal apoptosis, and suppress microglial activation while simultaneously inhibiting neuroinflammatory responses, thereby ameliorating cognitive dysfunction induced by chronic alcohol exposure in Table 2.

**TABLE 2:** The targets and specific mechanisms of ES active ingredients in treating KS.

Eleutherococcus senticosus components	The CNS target sites	The mechanism of action of KS
Eleutheroside B	Cortical neurons	1. Activate the PI3K/Akt signaling pathway 2. Inhibit p53 DNA-binding activity 3. Reduce oxidative stress in the brain 4. Decrease Aβ accumulation and neuronal apoptosis 5. Improve hippocampal synaptic plasticity
Eleutheroside E	Neuronal	1. Modulate the α7-nAChR-NMDAR signaling pathway
Triterpenoid saponins	Neurites and axons	1. Reduce β-amyloid-induced neural network damage by promoting the growth of neurites and axons
Flavonoid components	Neuronal in the ischemic brain region	1. Alleviate lipid peroxidation damage 2. Enhance the expression of Bcl-2 protein 3. Educe neuronal apoptosis 4. Activate the Keap1/Nrf2/HO-1 pathway
Polysaccharide active ingredients	Hippocampal neurons	1. Inhibit alcohol-induced hypoglycemia and neuronal inflammatory responses 2. Activate of the SIRT1/PGC-1α pathway 3. Improve mitochondrial damage 4. Reduce ROS production 5. Inhibit neuroinflammation
The aqueous extract of ES fruits	Microglial cells	1. Inhibit neuroinflammation 2. Inhibit the p38-CREB pathway-induced HO-1 expression 3. Activate the p38-CREB pathway 4. Promote the phosphorylation of the MAPK signaling pathway
Quercetin	Microglial cells	Promote the phosphorylation of the MAPK signaling pathway
ES extract	The gut microbiota	1. Reduce the abundance of pathogenic bacteria and increasing the abundance of probiotic bacteria 2. Increase the level of tight junction proteins 3. Inhibit the expression of inflammation in the colon 4. Promote the protein expression levels of BDNF and NF-κB 5. Decrease the relative protein expression level of nuclear factor IκBα in the hippocampus

Therefore, we hypothesize that ES possesses significant potential for treating KS. Its brain-penetrating components significantly inhibit neuronal apoptosis and microglial activation caused by long-term alcohol exposure, a risk factor for KS. Components such as flavonoids, polysaccharides, and glycosides may serve as the potential pharmacological basis for its therapeutic effects on KS. Compared to conventional thiamine direct supplementation therapy, ES not only treats KS by protecting the CNS but also achieves therapeutic effects through the gut-lung axis. After identifying the main active components of ES that exert therapeutic effects on KS, the development of novel targeted formulations will achieve more significant therapeutic outcomes.

We advocate for the collection of KS case data from the past 5 years. All cases should be jointly diagnosed using both Western and traditional Chinese medicine diagnostic criteria. A comprehensive description of the disease’s distribution across populations, regions, and time should be provided. Patients will be stratified and randomly assigned to blocks. Both healthcare providers and participants will be blinded to the group assignments. The intervention period will last for 8 weeks, during which the routine medication for underlying diseases (such as antihypertensive drugs) will be maintained, and the concurrent use of other nootropic agents will be prohibited. Follow up every 2 weeks to record medication adherence and adverse events (graded according to CTCAE 5.0 standards). After 8 weeks of treatment, use the MMSE score, Montreal Cognitive Assessment (MoCA), Rey Auditory Verbal Learning Test (RAVLT), Activities of Daily Living (ADL) scale, and TCM syndrome score table to evaluate the intervention effects of Acanthopanax on patients’ memory and cognitive impairments. Collect fecal, blood, and urine samples from patients before and at the 8th week of Acanthopanax treatment for microbiological and metabolomics studies. Use brain imaging techniques to observe and compare hippocampal volume and white matter integrity in patients. Apply appropriate statistical methods to analyze the collected data to determine whether the differences between the treatment and control groups are statistically significant. Meanwhile, any adverse reactions that may occur during the treatment process will be closely monitored to assess the safety and tolerability of ES Injection. Through this series of rigorous clinical research designs and evaluation methods, the aim is to provide a scientific basis for the clinical application of ES injection. Meanwhile, it lays the foundation for the research on novel targeted formulations of active ingredients in ES.

## References

[B1] Abrahao K. Salinas A. Lovinger D. (2017). Alcohol and the brain: Neuronal molecular targets, synapses, and circuits. *Neuron* 96 1223–1238. 10.1016/j.neuron.2017.10.032 29268093 PMC6566861

[B2] Ahmed S. Moni D. Sonawane K. Paek K. Shohael A. M. (2021). A comprehensive in silico exploration of pharmacological properties, bioactivities and COX-2 inhibitory potential of eleutheroside B from Eleutherococcus senticosus (Rupr. & Maxim.) Maxim. *J. Biomol. Struct. Dyn.* 39 6553–6566. 10.1080/07391102.2020.1803135 32757816

[B3] Alfonso-Loeches S. Pascual M. Gómez-Pinedo U. Pascual-Lucas M. Renau-Piqueras J. Guerri C. (2012). Toll-like receptor 4 participates in the myelin disruptions associated with chronic alcohol abuse. *Glia* 60 948–964. 10.1002/glia.22327 22431236

[B4] Arts N. Walvoort S. Kessels R. (2017). Korsakoff’s syndrome: A critical review. *Neuropsychiatr. Dis. Treat* 13 2875–2890. 10.2147/NDT.S130078 29225466 PMC5708199

[B5] Bâ A. (2017). Alcohol and thiamine deficiency trigger differential mitochondrial transition pore opening mediating cellular death. *Apoptosis* 22 741–752. 10.1007/s10495-017-1372-4 28417285

[B6] Bang J. Chung Y. Chung S. Lee H. Song E. Shin Y. (2015). Clinical effect of a polysaccharide-rich extract of Acanthopanax senticosus on alcohol hangover. *Pharmazie* 70 269–273. 10.1691/ph.2015.4786 26012258

[B7] Bansal L. Zinkus T. (2019). Osmotic demyelination syndrome in children. *Pediatr. Neurol.* 97 12–17. 10.1016/j.pediatrneurol.2019.03.018 31128892

[B8] Bhembre N. Bonthron C. Opazo P. (2023). Synaptic compensatory plasticity in Alzheimer’s disease. *J. Neurosci.* 43 6833–6840. 10.1523/JNEUROSCI.0379-23.2023 37821232 PMC10573755

[B9] Botta P. de Souza F. Sangrey T. De Schutter E. Valenzuela C. (2010). Alcohol excites cerebellar Golgi cells by inhibiting the Na+/K+ ATPase. *Neuropsychopharmacology* 35 1984–1996. 10.1038/npp.2010.76 20520600 PMC2904864

[B10] Burke M. Ptito M. Ervin F. Palmour R. (2015). Hippocampal neuron populations are reduced in vervet monkeys with fetal alcohol exposure. *Dev. Psychobiol.* 57 470–485. 10.1002/dev.21311 25913787 PMC4437182

[B11] Butterworth R. (1995). Pathophysiology of alcoholic brain damage: Synergistic effects of ethanol, thiamine deficiency and alcoholic liver disease. *Metab. Brain Dis.* 10 1–8. 10.1007/BF01991777 7596324

[B12] Byun K. Bayarsaikhan D. Bayarsaikhan E. Son M. Oh S. Lee J. (2014). Microglial AGE-albumin is critical in promoting alcohol-induced neurodegeneration in rats and humans. *PLoS One* 9:e104699. 10.1371/journal.pone.0104699 25140518 PMC4139297

[B13] Campanella S. Petit G. Maurage P. Kornreich C. Verbanck P. Noël X. (2009). Chronic alcoholism: Insights from neurophysiology. *Neurophysiol. Clin.* 39 191–207. 10.1016/j.neucli.2009.08.002 19853791

[B14] Chen Y. Chen H. Zhu W. (2023). Research progress and industrialization application of acanthopanax senticosu. *Pharmacy Today* 34, 312–320.

[B15] Cheng M. Yuan C. Ju Y. Liu Y. Shi B. Yang Y. (2024). Quercetin attenuates oxidative stress and apoptosis in brain tissue of APP/PS1 double transgenic AD mice by regulating Keap1/Nrf2/HO-1 pathway to improve cognitive impairment. *Behav. Neurol.* 2024:5698119. 10.1155/2024/5698119 39233848 PMC11374423

[B16] Chiarugi A. (2023). Glaucoma: Neuroprotection with NAD-based therapeutic interventions. *Trends Pharmacol. Sci.* 44 869–879. 10.1016/j.tips.2023.09.010 37880000

[B17] Cook C. Hallwood P. Thomson A. D. (1998). B Vitamin deficiency and neuropsychiatric syndromes in alcohol misuse. *Alcohol Alcohol* 33 317–336. 10.1093/oxfordjournals.alcalc.a008400 9719389

[B18] Cruz C. Meireles M. Silva S. (2017). Chronic ethanol intake induces partial microglial activation that is not reversed by long-term ethanol withdrawal in the rat hippocampal formation. *Neurotoxicology* 60 107–115. 10.1016/j.neuro.2017.04.005 28408342

[B19] Dai H. Jiu Y. Li X. Dai S. (2025). The effect of acanthopanax senticosus polysaccharide on hippocampal neuronal injury in sleep-deprived rats via regulation of the SIRT1/PGC-1α pathway. *Guiding J. Traditional Chin. Med. Pharm.* 10.13862/j.cn43-1446/r.2025.01.009

[B20] Darbinian N. Selzer M. (2022). Oligodendrocyte pathology in fetal alcohol spectrum disorders. *Neural Regen. Res.* 17 497–502. 10.4103/1673-5374.314294 34380877 PMC8504395

[B21] Deng F. Wang B. Shen Y. Jin X. Zhao H. (2020). Protective efficacy of eleutheroside B on cortical neuron injury induced by hypoxia/reoxygenation in SD rats and the impact on PI3K/Akt signaling pathway. *China Med. Pharm.* 10, 27–30. 10.3969/j.issn.2095-0616.2020.21.008

[B22] Deyama T. Nishibe S. Nakazawa Y. (2001). Constituents and pharmacological effects of Eucommia and Siberian ginseng. *Acta Pharmacol. Sin.* 22 1057–1070. 11749801

[B23] Ding M. Qu Y. Hu B. An H. (2022). Signal pathways in the treatment of Alzheimer’s disease with traditional Chinese medicine. *Biomed. Pharmacother.* 152:113208. 10.1016/j.biopha.2022.113208 35660246

[B24] Docherty J. (2025). Therapeutic potential of faecal microbiota transplantation for alcohol use disorder, a narrative synthesis. *Prog. Neuropsychopharmacol. Biol. Psychiatry* 138:111354. 10.1016/j.pnpbp.2025.111354 40185194

[B25] D’Orazi F. Zhao X. Wong R. Yoshimatsu T. (2016). Mismatch of synaptic patterns between neurons produced in regeneration and during development of the vertebrate retina. *Curr. Biol.* 26 2268–2279. 10.1016/j.cub.2016.06.063 27524481 PMC5534240

[B26] Egervari G. Siciliano C. Whiteley E. Ron D. (2021). Alcohol and the brain: From genes to circuits. *Trends Neurosci.* 44 1004–1015. 10.1016/j.tins.2021.09.006 34702580 PMC8616825

[B27] Fu J. Gao X. Lu Y. Lu F. Wang Y. Chen P. (2024). Integrated proteomics and metabolomics reveals metabolism disorders in the α-syn mice and potential therapeutic effect of Acanthopanax senticosus extracts. *J. Ethnopharmacol.* 318:116878. 10.1016/j.jep.2023.116878 37419226

[B28] García-Baos A. Alegre-Zurano L. Cantacorps L. Martín-Sánchez A. Valverde O. (2021). Role of cannabinoids in alcohol-induced neuroinflammation. *Prog. Neuropsychopharmacol. Biol. Psychiatry* 104:110054. 10.1016/j.pnpbp.2020.110054 32758518

[B29] Ge Y. Tohda C. Zhu S. He Y. Yoshimatsu K. Komatsu K. (2016). Effects of oleanane-type triterpene saponins from the leaves of eleutherococcus senticosus in an axonal outgrowth assay. *J. Nat. Prod.* 79 1834–1841. 10.1021/acs.jnatprod.6b00329 27400231

[B30] Gerontakos S. Taylor A. Avdeeva A. Shikova V. Pozharitskaya O. Casteleijn D. (2021). Findings of Russian literature on the clinical application of Eleutherococcus senticosus (Rupr. & Maxim.): A narrative review. *J. Ethnopharmacol.* 278:114274. 10.1016/j.jep.2021.114274 34087398

[B31] Golub H. Zhou Q. Zucker H. McMullen M. Kokiko-Cochran O. Ro E. (2015). Chronic alcohol exposure is associated with decreased neurogenesis, aberrant integration of newborn neurons, and cognitive dysfunction in female mice. *Alcohol Clin. Exp. Res.* 39 1967–1977. 10.1111/acer.12843 26365148 PMC4592440

[B32] Guergues J. Wohlfahrt J. Zhang P. Liu B. Stevens S. (2020). Deep proteome profiling reveals novel pathways associated with pro-inflammatory and alcohol-induced microglial activation phenotypes. *J. Proteomics* 220:103753. 10.1016/j.jprot.2020.103753 32200115 PMC7334825

[B33] Guizzetti M. Costa L. (2005). Disruption of cholesterol homeostasis in the developing brain as a potential mechanism contributing to the developmental neurotoxicity of ethanol: An hypothesis. *Med. Hypotheses* 64 563–567. 10.1016/j.mehy.2004.05.019 15617867

[B34] Henriques J. Portugal C. Canedo T. Relvas J. Summavielle T. Socodato R. (2018). Microglia and alcohol meet at the crossroads: Microglia as critical modulators of alcohol neurotoxicity. *Toxicol. Lett.* 283 21–31. 10.1016/j.toxlet.2017.11.002 29129797

[B35] Huang D. Hu Z. Yu Z. (2013). Eleutheroside B or E enhances learning and memory in experimentally aged rats. *Neural Regen. Res.* 8 1103–1112. 10.3969/j.issn.1673-5374.2013.12.005 25206404 PMC4145894

[B36] Huang D. Li M. Qiao Z. Zhou H. Cai Y. Li X. (2024). Effects of adolescent alcohol exposure on oligodendrocyte lineage cells and myelination in mice: Age and subregion differences. *IBRO Neurosci. Rep.* 17 220–234. 10.1016/j.ibneur.2024.06.006 39282551 PMC11401168

[B37] Hyatt H. Ozdemir M. Bomkamp M. Powers S. (2022). Activation of calpain contributes to mechanical ventilation-induced depression of protein synthesis in diaphragm muscle. *Cells* 11:1028. 10.3390/cells11061028 35326479 PMC8947683

[B38] Im P. Wright N. Yang L. Chan K. Chen Y. Guo Y. (2023). Alcohol consumption and risks of more than 200 diseases in Chinese men. *Nat. Med.* 29 1476–1486. 10.1038/s41591-023-02383-8 37291211 PMC10287564

[B39] Jin M. Park S. Kim Y. Park G. Lee S. (2013). Acanthopanax senticosus exerts neuroprotective effects through HO-1 signaling in hippocampal and microglial cells. *Environ. Toxicol. Pharmacol.* 35 335–346. 10.1016/j.etap.2013.01.004 23395777

[B40] Kalueff A. Stewart A. Gerlai R. (2014). Zebrafish as an emerging model for studying complex brain disorders. *Trends Pharmacol. Sci.* 35 63–75. 10.1016/j.tips.2013.12.002 24412421 PMC3913794

[B41] Khan M. Chang S. (2023). Alcohol and the brain-gut axis: The involvement of microglia and enteric glia in the process of neuro-enteric inflammation. *Cells* 12:2475. 10.3390/cells12202475 37887319 PMC10605902

[B42] Klann E. Dissanayake U. Gurrala A. Farrer M. Wagle Shukla A. Ramirez-Zamora A. (2021). The gut-brain axis and its relation to Parkinson’s disease: A review. *Front. Aging Neurosci.* 13:782082. 10.3389/fnagi.2021.782082 35069178 PMC8776990

[B43] Koike H. Sobue G. (2006). Alcoholic neuropathy. *Curr. Opin. Neurol.* 19 481–486. 10.1097/01.wco.0000245371.89941.eb 16969158

[B44] Kong X. Lyu W. Lin X. Lin C. Feng H. Xu L. (2024). Itaconate alleviates anesthesia/surgery-induced cognitive impairment by activating a Nrf2-dependent anti-neuroinflammation and neurogenesis via gut-brain axis. *J. Neuroinflammation* 21:104. 10.1186/s12974-024-03103-w 38649932 PMC11034021

[B45] Kopelman M. Thomson A. Guerrini I. Marshall E. (2009). The Korsakoff syndrome: Clinical aspects, psychology and treatment. *Alcohol Alcohol* 44 148–154. 10.1093/alcalc/agn118 19151162

[B46] Kuhn S. Gritti L. Crooks D. Dombrowski Y. (2019). Oligodendrocytes in development, myelin generation and beyond. *Cells* 8:1424. 10.3390/cells8111424 31726662 PMC6912544

[B47] Kunkle B. W. Grenier-Boley B. Sims R. Bis J. C. Damotte V. Naj A. C. (2019). Genetic meta-analysis of diagnosed Alzheimer’s disease identifies new risk loci and implicates Aβ, tau, immunity and lipid processing. *Nat. Genet.* 51 414–430. 10.1038/s41588-019-0358-2 30820047 PMC6463297

[B48] Lee S. Park H. Jeon S. Kim E. Lee H. Kim H. (2017). Cognitive ameliorating effect of acanthopanax koreanum against scopolamine-induced memory impairment in mice. *Phytother. Res.* 31 425–432. 10.1002/ptr.5764 28164395

[B49] Li J. Wang H. Liu D. Li X. He L. Pan J. (2023). CB2R activation ameliorates late adolescent chronic alcohol exposure-induced anxiety-like behaviors during withdrawal by preventing morphological changes and suppressing NLRP3 inflammasome activation in prefrontal cortex microglia in mice. *Brain Behav. Immun.* 110 60–79. 10.1016/j.bbi.2023.02.001 36754245

[B50] Li Q. Liu D. Pan F. Ho C. Ho R. (2019). Ethanol exposure induces microglia activation and neuroinflammation through TLR4 activation and SENP6 modulation in the adolescent rat hippocampus. *Neural Plast.* 2019:1648736. 10.1155/2019/1648736 31781182 PMC6874951

[B51] Li Q. Zhao Y. Guo H. Li Q. Yan C. Li Y. (2023). Impaired lipophagy induced-microglial lipid droplets accumulation contributes to the buildup of TREM1 in diabetes-associated cognitive impairment. *Autophagy* 19 2639–2656. 10.1080/15548627.2023.2213984 37204119 PMC10472854

[B52] Li X. Chen L. Kumar G. Zhang S. Zhong Q. Zhang H. (2022). Therapeutic interventions of gut-brain axis as novel strategies for treatment of alcohol use disorder associated cognitive and mood dysfunction. *Front. Neurosci.* 16:820106. 10.3389/fnins.2022.820106 35185459 PMC8847450

[B53] Li X. Liu J. Boreland A. Kapadia S. Zhang S. Stillitano A. (2024). Polygenic risk for alcohol use disorder affects cellular responses to ethanol exposure in a human microglial cell model. *Sci. Adv.* 10:eado5820. 10.1126/sciadv.ado5820 39514655 PMC11546823

[B54] Liu D. Li J. Rong X. Li J. Peng Y. Shen Q. (2022). Cdk5 promotes mitochondrial fission via Drp1 phosphorylation at S616 in chronic ethanol exposure-induced cognitive impairment. *Mol. Neurobiol.* 59 7075–7094. 10.1007/s12035-022-03008-w 36083519

[B55] Liu S. Li X. Zhang S. Yang Z. Wang K. Lu F. (2018). Acanthopanax senticosus protects structure and function of mesencephalic mitochondria in a mouse model of Parkinson’s disease. *Chin. J. Integr. Med.* 24 835–843. 10.1007/s11655-018-2935-5 30090975

[B56] Liu Y. Huang Q. Cao J. (2013). Protective effect of acanthopanacis senticosi polysaccharides on H2O2-induecd cell apoptosis of primary cultured rat hippocampal neurons. *Chinese J. Clin. Neurosurg.* 18, 681–683. doi: CNKI:SUN:ZGLC.0.2013-11-016

[B57] Lu R. Voigt R. Zhang Y. Kato I. Xia Y. Forsyth C. (2017). Alcohol injury damages intestinal stem cells. *Alcohol. Clin. Exp. Res.* 41 727–734. 10.1111/acer.13351 28195397 PMC5378625

[B58] Lu S. Ge Q. Yang M. Zhuang Y. Xu X. Niu F. (2024). Decoupling the mutual promotion of inflammation and oxidative stress mitigates cognitive decline and depression-like behavior in rmTBI mice by promoting myelin renewal and neuronal survival. *Biomed. Pharmacother.* 173:116419. 10.1016/j.biopha.2024.116419 38479178

[B59] Lu T. Hanumaihgari P. Hsu E. Agarwal A. Kawaguchi R. Calabresi P. (2023). Norepinephrine modulates calcium dynamics in cortical oligodendrocyte precursor cells promoting proliferation during arousal in mice. *Nat. Neurosci.* 26 1739–1750. 10.1038/s41593-023-01426-0 37697112 PMC10630072

[B60] Lu X. Xiao-Qing C. (2019). Eleutheroside E attenuates isoflurane-induced cognitive dysfunction by regulating the α7-nAChR-NMDAR pathway. *Neuroreport* 30 188–194. 10.1097/WNR.0000000000001182 30585907

[B61] Ma M. Han L. Ren T. Sun X. Hua X. (2025). Research progress on the pharmacological effects of acanthopanax senticosus. *J. Jilin Med. Univer.* 10.13845/j.cnki.issn1673-2995.20250205.001

[B62] Malpetti M. Cope T. Street D. Jones P. Hezemans F. Mak E. (2023). Microglial activation in the frontal cortex predicts cognitive decline in frontotemporal dementia. *Brain* 146 3221–3231. 10.1093/brain/awad078 36883644 PMC10393407

[B63] Marizzoni M. Mirabelli P. Mombelli E. Coppola L. Festari C. Lopizzo N. (2023). A peripheral signature of Alzheimer’s disease featuring microbiota-gut-brain axis markers. *Alzheimers Res. Ther.* 15:101. 10.1186/s13195-023-01218-5 37254223 PMC10230724

[B64] Marshall S. Geil C. Nixon K. (2016). Prior binge ethanol exposure potentiates the microglial response in a model of alcohol-induced neurodegeneration. *Brain Sci.* 6:16. 10.3390/brainsci6020016 27240410 PMC4931493

[B65] Martin P. Singleton C. Hiller-Sturmhöfel S. (2003). The role of thiamine deficiency in alcoholic brain disease. *Alcohol. Res. Health* 27 134–142. 10.1093/alcalc/agg021 15303623 PMC6668887

[B66] McCarthy G. Farris S. Blednov Y. Harris R. Mayfield R. (2018). Microglial-specific transcriptome changes following chronic alcohol consumption. *Neuropharmacology* 128 416–424. 10.1016/j.neuropharm.2017.10.035 29101021 PMC5990017

[B67] McKee M. (1999). Alcohol in Russia. *Alcohol Alcohol* 34 824–829. 10.1093/alcalc/34.6.824 10659717

[B68] Mira R. Tapia-Rojas C. Pérez M. Jara C. Vergara E. Quintanilla R. (2019). Alcohol impairs hippocampal function: From NMDA receptor synaptic transmission to mitochondrial function. *Drug. Alcohol Depend.* 205:107628. 10.1016/j.drugalcdep.2019.107628 31683244

[B69] Muley A. Fernandez R. Green H. Muley P. (2022). Effect of thiamine supplementation on glycaemic outcomes in adults with type 2 diabetes: A systematic review and meta-analysis. *BMJ Open* 12:e059834. 10.1136/bmjopen-2021-059834 36008064 PMC9422810

[B70] Neufeld M. Rehm J. (2013). Alcohol consumption and mortality in Russia since 2000: Are there any changes following the alcohol policy changes starting in 2006? *Alcohol Alcohol.* 48 222–230. 10.1093/alcalc/ags134 23299570

[B71] Nutt D. Hayes A. Fonville L. Zafar R. Palmer E. Paterson L. (2021). Alcohol and the brain. *Nutrients* 13:3938. 10.3390/nu13113938 34836193 PMC8625009

[B72] Oudman E. Nijboer T. Postma A. Wijnia J. Van der Stigchel S. (2015). Procedural learning and memory rehabilitation in korsakoff’s syndrome - a review of the literature. *Neuropsychol. Rev.* 25 134–148. 10.1007/s11065-015-9288-7 26047664 PMC4464729

[B73] Pan W. Zhao J. Wu J. Xu D. Meng X. Jiang P. (2023). Dimethyl itaconate ameliorates cognitive impairment induced by a high-fat diet via the gut-brain axis in mice. *Microbiome* 11:30. 10.1186/s40168-023-01471-8 36810115 PMC9942412

[B74] Pandey S. Kyzar E. Zhang H. (2017). Epigenetic basis of the dark side of alcohol addiction. *Neuropharmacology* 122 74–84. 10.1016/j.neuropharm.2017.02.002 28174112 PMC5479721

[B75] Paterson C. Gutierrez M. Coopersmith C. Ford M. (2023). Impact of chronic alcohol exposure on conventional and regulatory murine T cell subsets. *Front. Immunol.* 14:1142614. 10.3389/fimmu.2023.1142614 37006296 PMC10063870

[B76] Paul I. Tsang B. Gerlai R. (2020). Short exposure to moderate concentration of alcohol during embryonic development does not alter gross morphology in *Zebrafish*. *Zebrafish* 17 253–260. 10.1089/zeb.2020.1872 32493176

[B77] Pei J. Zhang C. Zhang Q. Yu H. Yuan H. Guo Y. (2024). Probiotics alleviate chronic ethanol exposure-induced anxiety-like behavior and hippocampal neuroinflammation in male mice through gut microbiota-derived extracellular vesicles. *J. Nanobiotechnol.* 22:730. 10.1186/s12951-024-03017-y 39578835 PMC11585232

[B78] Pizarro N. Kossatz E. González P. Gamero A. Veza E. Fernández C. (2021). Sex-specific effects of synbiotic exposure in mice on addictive-like behavioral alterations induced by chronic alcohol intake are associated with changes in specific gut bacterial taxa and brain tryptophan metabolism. *Front. Nutr.* 8:750333. 10.3389/fnut.2021.750333 34901109 PMC8662823

[B79] Poh L. Kang S. Baik S. Ng G. She D. Balaganapathy P. (2019). Evidence that NLRC4 inflammasome mediates apoptotic and pyroptotic microglial death following ischemic stroke. *Brain Behav. Immun.* 75 34–47. 10.1016/j.bbi.2018.09.001 30195027

[B80] Popa I. Drăgoi A. Trifu S. Cristea M. (2021). Korsakoff syndrome: An overlook (Review). *Exp. Ther. Med.* 22:1132. 10.3892/etm.2021.10566 34466144 PMC8383329

[B81] Rajesh V. Mridhulmohan M. Jayaseelan S. Sivakumar P. Ganesan V. (2018). Mefenamic acid attenuates chronic alcohol induced cognitive impairment in zebrafish: Possible role of cholinergic pathway. *Neurochem. Res.* 43 1392–1404. 10.1007/s11064-018-2554-3 29796737

[B82] Rehm J. Shield K. (2019). Global burden of disease and the impact of mental and addictive disorders. *Curr. Psychiatry Rep.* 21:10. 10.1007/s11920-019-0997-0 30729322

[B83] Samantaray S. Knaryan V. Patel K. Mulholland P. Becker H. Banik N. (2015). Chronic intermittent ethanol induced axon and myelin degeneration is attenuated by calpain inhibition. *Brain Res.* 1622 7–21. 10.1016/j.brainres.2015.06.014 26100335 PMC4562802

[B84] Savage L. Candon P. Hohmann H. (2000). Alcohol-induced brain pathology and behavioral dysfunction: Using an animal model to examine sex differences. *Alcohol Clin. Exp. Res.* 24 465–475. 10.1111/j.1530-0277.2000.tb02013.x 10798582

[B85] Schwarzinger M. Pollock B. Hasan O. Dufouil C. Rehm J. (2018). Contribution of alcohol use disorders to the burden of dementia in France 2008-13: A nationwide retrospective cohort study. *Lancet Public Health* 3 e124–e132. 10.1016/S2468-2667(18)30022-7 29475810

[B86] Singh A. Naidu P. Kulkarni S. (2003). Reversal of aging and chronic ethanol-induced cognitive dysfunction by quercetin a bioflavonoid. *Free Radic. Res.* 37 1245–1252. 10.1080/10715760310001616014 14703737

[B87] Song C. Li S. Duan F. Liu M. Shan S. Ju T. (2022). The therapeutic effect of acanthopanax senticosus components on radiation-induced brain Injury based on the pharmacokinetics and neurotransmitters. *Molecules* 27:1106. 10.3390/molecules27031106 35164373 PMC8839712

[B88] Song C. Yin Y. Qin Y. Li T. Zeng D. Ju T. (2023). Acanthopanax senticosus extract alleviates radiation-induced learning and memory impairment based on neurotransmitter-gut microbiota communication. *CNS Neurosci. Ther.* 29 129–145. 10.1111/cns.14134 36971202 PMC10314102

[B89] Song Y. Wang Z. Feng X. Deng X. Zhu J. (2016). Simultaneous determination and pharmacokinetics of four triterpenoids by ultra high performance liquid chromatography with tandem mass spectrometry after the oral administration of Acanthopanax sessiliflorus (Rupr. et Maxim) Seem extract. *J. Sep. Sci.* 39 2229–2237. 10.1002/jssc.201501350 27324351

[B90] Suk K. (2007). Microglial signal transduction as a target of alcohol action in the brain. *Curr. Neurovasc. Res.* 4 131–142. 10.2174/156720207780637261 17504211

[B91] Sun H. Lv H. Zhang Y. Wang X. Bi K. Cao H. (2007). Pharmacokinetics of isofraxidin in rat plasma after oral administration of the extract of Acanthopanax senticosus using HPLC with solid phase extraction method. *Chem. Pharm. Bull.* 55 1291–1295. 10.1248/cpb.55.1291 17827750

[B92] Tong Z. Feng W. Cao G. Sun G. (2025). Study on medicinal value and resource evaluation of genuine regional medicine acanthopanax senticosus from Heilongjiang Province. *Chin. J. Library Information Sci. Traditional Chinese Med.* 49, 29–33.

[B93] Tripathi R. Gupta R. Sahu M. Srivastava D. Das A. Ambasta R. (2022). Free radical biology in neurological manifestations: Mechanisms to therapeutics interventions. *Environ. Sci. Pollut. Res. Int.* 29 62160–62207. 10.1007/s11356-021-16693-2 34617231

[B94] Tufvesson-Alm M. Shevchouk O. Jerlhag E. (2022). Insight into the role of the gut-brain axis in alcohol-related responses: Emphasis on GLP-1, amylin, and ghrelin. *Front. Psychiatry* 13:1092828. 10.3389/fpsyt.2022.1092828 36699502 PMC9868418

[B95] Tyler C. Allan A. (2014). Prenatal alcohol exposure alters expression of neurogenesis-related genes in an ex vivo cell culture model. *Alcohol* 48 483–492. 10.1016/j.alcohol.2014.06.001 24954023 PMC4096774

[B96] van Dam M. van Meijel B. Postma A. Oudman E. (2020). Health problems and care needs in patients with Korsakoff’s syndrome: A systematic review. *J. Psychiatr. Ment. Health Nurs.* 27 460–481. 10.1111/jpm.12587 31876326

[B97] Waddell J. McKenna M. Kristian T. (2022). Brain ethanol metabolism and mitochondria. *Curr. Top. Biochem. Res.* 23 1–13. 36873619 PMC9980429

[B98] Waldmann T. Dubois S. Miljkovic M. Conlon K. C. (2020). IL-15 in the combination immunotherapy of cancer. *Front. Immunol.* 11:868. 10.3389/fimmu.2020.00868 32508818 PMC7248178

[B99] Walter K. Ricketts D. Presswood B. Smith S. Mooney S. (2023). Prenatal alcohol exposure causes persistent microglial activation and age- and sex- specific effects on cognition and metabolic outcomes in an Alzheimer’s Disease mouse model. *Am. J. Drug Alcohol. Abuse* 49 302–320. 10.1080/00952990.2022.2119571 36194703 PMC11040461

[B100] Wang Q. Guo L. Zhang B. Cai Y. Li T. (2014). Control the quality standard of Acanthopanax. *Chinese J. Clin. Pharmacol.* 30, 553–555. 10.13699/j.cnki.1001-6821.2014.06.026

[B101] Wang R. Sun Y. Wang M. Li H. Liu S. Liu Z. (2023). Therapeutic effect of Eleutherococcus senticosus (Rupr. & Maxim.) Maxim. leaves on ischemic stroke via the microbiota-gut-brain axis. *Phytotherapy Res.* 37 4801–4818. 10.1002/ptr.7947 37518502

[B102] Wang S. Chen Y. Chen S. Lee C. Cheng C. (2020). Alcohol addiction, gut microbiota, and alcoholism treatment: A review. *Int. J. Mol. Sci.* 21:6413. 10.3390/ijms21176413 32899236 PMC7504034

[B103] Wang W. Lu J. Ma F. Wang J. Ma Y. Hua X. (2022). Protective effect of acanthopanax senticosus extract on SH-SY5Y cells after oxygen and glucose deprivation reperfusion injury. *Modernization Traditional Chin. Med. Materia Medica World Sci. Technol.* 24, 1772–1780. 10.11842/wst.20211115008

[B104] Wang Y. Liu S. Wang R. Shi L. Liu Z. Liu Z. (2020). Study on the therapeutic material basis and effect of Acanthopanax senticosus (Rupr. et Maxim.) Harms leaves in the treatment of ischemic stroke by PK-PD analysis based on online microdialysis-LC-MS/MS method. *Food Funct.* 11 2005–2016. 10.1039/c9fo02475a 32077871

[B105] Wang Y. Meng Y. Zhai C. Wang M. Avula B. Yuk J. (2019). The chemical characterization of eleutherococcus senticosus and Ci-wu-jia tea using UHPLC-UV-QTOF/MS. *Int. J. Mol. Sci.* 20:475. 10.3390/ijms20030475 30678313 PMC6387334

[B106] Wolstenholme J. Duong N. Brocato E. Bajaj J. (2024). Gut-liver-brain axis and alcohol use disorder: Treatment potential of fecal microbiota transplantation. *Alcohol Res.* 44:1. 10.35946/arcr.v44.1.01 38322428 PMC10843328

[B107] Wong E. Lutz N. Hogan V. Lamantia C. McMurray H. Myers J. (2018). Developmental alcohol exposure impairs synaptic plasticity without overtly altering microglial function in mouse visual cortex. *Brain Behav. Immun.* 67 257–278. 10.1016/j.bbi.2017.09.003 28918081 PMC5696045

[B108] Wu F. An L. Huang J. Lu F. Ge S. Su X. (2025). Research progress on the chemical compositions and pharmacological effects of Ciwujia (acanthopanacis senticosi radix et rhizoma seu caulis). *Guiding J. Traditional Chinese Med. Pharm.* 10.13862/j.cn43-1446/r.2025.02.020

[B109] Wu F. Zhu J. Li G. Wang J. Veeraraghavan V. Krishna Mohan S. (2019). Biologically synthesized green gold nanoparticles from Siberian ginseng induce growth-inhibitory effect on melanoma cells (B16). *Artif. Cells Nanomed. Biotechnol.* 47 3297–3305. 10.1080/21691401.2019.1647224 31379212

[B110] Wu Q. Li P. Li X. Ma L. Chen K. Man S. (2024). Pueraria Extract ameliorates alcoholic liver disease via the liver-gut-brain axis: Focus on restoring the intestinal barrier and inhibiting alcohol metabolism. *J. Agric Food Chem.* 72 24449–24462. 10.1021/acs.jafc.4c05365 39445550

[B111] Xie Y. Zhang B. Zhang Y. (2015). Protective effects of Acanthopanax polysaccharides on cerebral ischemia-reperfusion injury and its mechanisms. *Int. J. Biol. Macromol.* 72 946–950. 10.1016/j.ijbiomac.2014.09.055 25451748

[B112] Yamauchi Y. Ge Y. Yoshimatsu K. Komastu K. Kuboyama T. Yang X. (2019). Memory enhancement by oral administration of extract of Eleutherococcus senticosus leaves and active compounds transferred in the brain. *Nutrients* 11:1142. 10.3390/nu11051142 31121888 PMC6567285

[B113] Yan T. Zhao Y. Jiang Z. Chen J. (2022). Acetaldehyde induces cytotoxicity via triggering mitochondrial dysfunction and overactive mitophagy. *Mol. Neurobiol.* 59 3933–3946. 10.1007/s12035-022-02828-0 35438433

[B114] Yang F. Wei J. Shen M. Ding Y. Lu Y. Ishaq H. (2021). Integrated analyses of the gut microbiota, intestinal permeability, and serum metabolome phenotype in rats with alcohol withdrawal syndrome. *Appl. Environ. Microbiol.* 87:e0083421. 10.1128/AEM.00834-21 34190609 PMC8388829

[B115] Zhang L. Li J. Li C. Wu Y. Liu S. Li Q. (2025). Role of microglial mitophagy in alleviating postoperative cognitive dysfunction: A mechanistic study. *Mol. Neurobiol.* 62 2376–2395. 10.1007/s12035-024-04405-z 39110392

[B116] Zhang Z. Wu Y. Shi D. Jiang C. Cao H. Jiang F. (2024). Acanthopanax senticosus improves cognitive impairment in Alzheimer’s disease by promoting the phosphorylation of the MAPK signaling pathway. *Front. Immunol.* 15:1383464. 10.3389/fimmu.2024.1383464 38545117 PMC10965608

[B117] Zhou A. Song B. Fu C. Baranenko D. Wang E. Li F. (2018). Acanthopanax senticosus reduces brain injury in mice exposed to low linear energy transfer radiation. *Biomed. Pharmacother.* 99 781–790. 10.1016/j.biopha.2018.01.001 29710476

[B118] Zhou Y. Cheng C. Baranenko D. Wang J. Li Y. Lu W. (2018). Effects of acanthopanax senticosus on brain injury induced by simulated spatial radiation in mouse model based on pharmacokinetics and comparative proteomics. *Int. J. Mol. Sci.* 19:159. 10.3390/ijms19010159 29342911 PMC5796108

[B119] Zhou Y. Ren Y. Li X. Cai M. Li H. Ding W. (2023). MS/MS molecular networking-guided in-depth profiling of triterpenoid saponins from the fruit of Eleutherococcus senticosus and their neuroprotectivity evaluation. *Phytochem. Anal.* 34 209–224. 10.1002/pca.3198 36529143

[B120] Zhu Y. Shi X. Yu Y. (2012). Protective effect of total flavones of acanthpanacis senticosi on ischemia inyury in cultured primary cortex neurons. *J. Mudanjiang Med. Univer.* 33, 3–5. 10.13799/j.cnki.mdjyxyxb.2012.02.018

[B121] Zhuo Y. Fu X. Jiang Q. Lai Y. Gu Y. Fang S. (2023). Systems pharmacology-based mechanism exploration of Acanthopanax senticosusin for Alzheimer’s disease using UPLC-Q-TOF-MS, network analysis, and experimental validation. *Eur. J. Pharmacol.* 954:175895. 10.1016/j.ejphar.2023.175895 37422122

